# Wide-range Vacuum Measurements from MWNT Field Emitters Grown Directly on Stainless Steel Substrates

**DOI:** 10.1186/s11671-015-1207-6

**Published:** 2016-01-06

**Authors:** Jian Zhang, Detian Li, Yangyang Zhao, Yongjun Cheng, Changkun Dong

**Affiliations:** Institute of Micro-nano Structures and Optoelectronics, Wenzhou University, Chashan University Town, Wenzhou China; Science and Technology on Vacuum Technology and Physics Laboratory, Lanzhou Institution of Physics, Lanzhou, China

**Keywords:** Field emission, Carbon nanotube, Ionization gauge, Ultra-high vacuum, Modulator

## Abstract

The field emission properties and the vacuum measurement application are investigated from the multi-walled carbon nanotubes (MWNTs) grown directly on catalytic stainless steel substrates. The MWNT emitters present excellent emission properties after the acid treatment of the substrate. The MWNT gauge is able to work down to the extreme-high vacuum (XHV) range with linear measurement performance in wide range from 10^−11^ to 10^−6^ Torr. A modulating grid is attempted with improved gauge sensitivity. The extension of the lower pressure limit is attributed largely to low outgassing effect due to direct growth of MWNTs and justified design of the electron source.

**Electronic supplementary material:**

The online version of this article (doi:10.1186/s11671-015-1207-6) contains supplementary material, which is available to authorized users.

## Background

The electron field emission has potential to overcome thermionic emission related problems [1–4] for merits such as room-temperature operation, low power consumption, and quick response capability. Carbon nanotubes (CNTs) possess unique electrical, chemical, and mechanical as well as structural properties and are regarded as the most promising field emission material with low emission fields and good vacuum behaviors [5–14]. The “cold” cathode operation could help to reduce the outgassings, benefiting greatly the ultra-high vacuum/extreme-high vacuum (UHV/XHV) measurement. Many investigations have been conducted to employ the CNT field emission in ionization gauge applications. Murakami et al. presented a Bayard-Alpert type CNT field emission gauge and tested in 10^−6^ to 10^−4^ Torr range [15]. In 2004, Dong and Myneni developed an extractor type field emission gauge based on the MWNTs grown on Ni alloy. The gauge presented excellent measurement linearity from 10^−10^ to 10^−6^ Torr with the sensitivity of about 3 Torr^−1^ [16, 17]. Huang et al. replaced thermionic filament of the Bayard-Alpert gauge (BAG) by a line-type CNT cathode, and the gauge performances were studied from 10^−4^ to 10^−7^ Torr with the sensitivity of 3.6 Torr^−1^ [18]. Suto et al. developed the CNT electron source by screen printing and studied its ionization gauge application [19].The gauge responded linearly from 10^−8^ to 10^−4^ Torr with the sensitivity of 13 Torr^−1^, close to the commercial gauge sensitivity. New gauge designs and structural modulations were also attempted. Sheng et al. constructed a saddle field gauge with a simple ring anode. The gauge was tested from 10^−5^ to 10^−3^ Pa with improved sensitivity of 1.7 Pa^−1^ [20]. A simple triode type of CNT ionization gauge was investigated by several groups, but there were no UHV/XHV measurement results [21–25].

The applications of CNT field emission in pressure measurement have not been well established. Some technical issues, including the outgassings due to the breaking off of the film components and the gas desorption from the CNT cathode, restrict the UHV/XHV applications. In this work, MWNTs are grown directly on the stainless steel substrate (S.S.) without extra catalyst layer by thermal chemical vapor deposition (CVD). Improved field emission performances are presented from the MWNT emitters after anodizing the substrate. The all mechanical assembly MWNT field emission cathode is constructed with low emission fields, high electron transmittances over the gate, and long-term stability. The ionization gauge behaviors based on the MWNT source are investigated with excellent measurement linearity from 10^−11^ to 10^−6^ Torr.

## Methods

The MWNT film is grown directly on the 304 S.S. without extra catalyst layer by the CVD technique from C_2_H_2_/Ar source gases at 750 °C. To improve the field emission properties, the S.S. substrate is anodized in the 0.3 mol L^−1^ oxalic acid solution. The field emission properties are compared for regular and anodized MWNT emitters from the diode setup. By screwing the parts together without any adhesive material, MWNT electron source is developed from totally mechanical assembly with features of low outgassing rates, good emission stability, and the feasibility to replace the MWNT cathode. The MWNT field emission ionization gauge is constructed upon the extractor gauge. The gauge performances are investigated inside a turbo vacuum system with 10^−11^ Torr background vacuum, and the pressure is calibrated against a commercial IE 514 extractor gauge. The measurement linearities are investigated for N_2_, H_2_, and O_2_, respectively, in wide pressure ranges. Keithley multimeters are used to supply the operation potentials and measure ion and electron currents.

## Results and Discussion

**Fig. 1:**
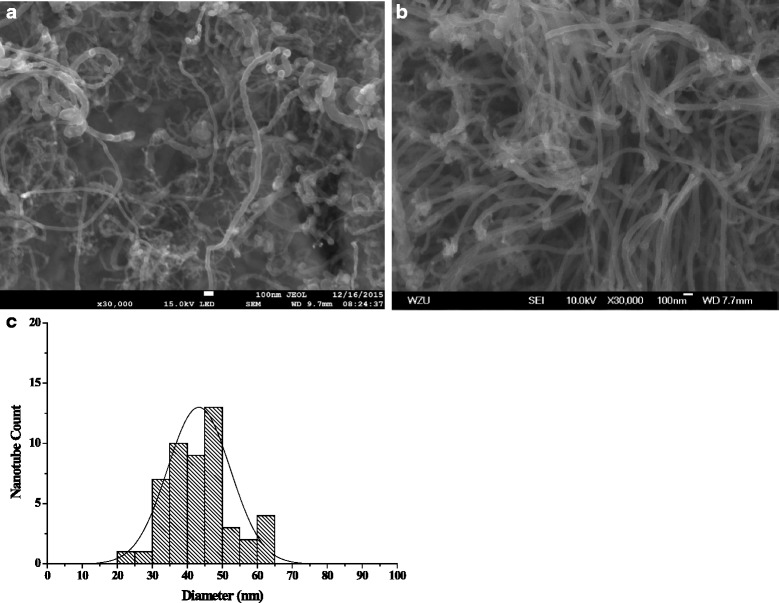
SEM images of MWNT emitters grown on the stainless steel substrate. **a** Regular sample. **b** After substrate anodization. **c** Histograms of the nanotubes diameter distribution corresponding to (**b**)

Figure 1a and b shows SEM images of the randomly oriented MWNTs grown on regular and anodized S.S. substrates, respectively. It is clear that the anodization process improves the CNT diameter uniformity, the density, and the straightness significantly. The average tube diameter is also smaller. According to the histogram plot of Fig. 1c counting on the nanotube diameters for Fig. 1b, the MWNTs of diameters in 30–50 nm account for 80 %. The analysis of the surface morphology shows that more uniform and higher density nano-scale pores appear after anodization comparing with the regular substrate, favoring the CNT growth, as shown in AI-1 of Additional file 1.

**Fig. 2:**
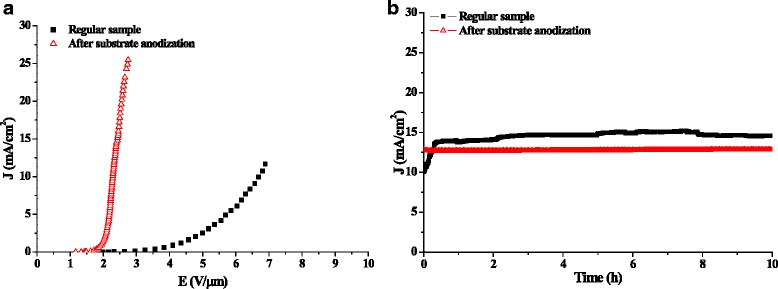
Emission improvements after the substrate anodization. **a** Emission current density-field curves. **b** Emission stabilities tested in 10^−8^ Torr

Field emission J-E and stability behaviors of MWNT films are tested, as shown in Fig. 2. After the anodization, the turn-on field drops from 2.2 to 1.4 V μm^−1^, and the threshold field drops from 6.68 to 2.28 V μm^−1^. Meanwhile, the emission under the current density of 12 mA cm^−2^ is more stable with the fluctuation of less than ±0.5 % in 10^−8^ Torr. The significant reductions of the emission fields are probably related to the decrease of the MWNT diameter and the increase of emission sites, where better straightness of the MWNT emitters are regarded as the key factor for the excellent emission stability after the anodization.

**Fig. 3:**
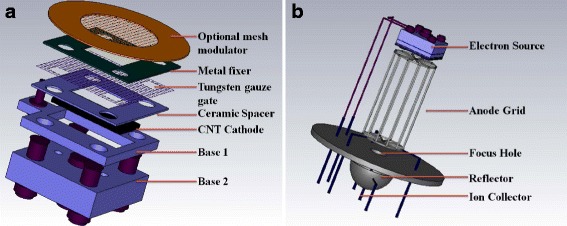
**a** Schematic drawing of the electron source with an optional mesh modulator. **b** Illustration of the MWNT ionization gauge with the spacing between the electron source and the anode of 2 mm

**Fig. 4:**
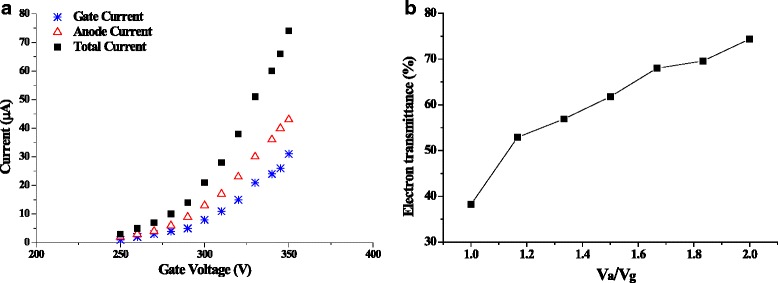
Emission characteristics of the electron source with the MWNT emission area of 20 mm^2^ and the cathode-gate spacing of 100 μm. **a** I-V curves under cathode-anode spacing of 2 mm and anode voltage of 430 V. **b** Electron transmittances over the gate grid with the increase of *V*_a_/*V*_g_ ratios

The structure of the MWNT electron source is illustrated in Fig. 3a. The extraction gate of 92.2 % transmittance tungsten gauze is separated from the MWNT cathode by a 100 μm aluminum oxide ceramic plate. An optional mesh modulator, which adjusts the electron energy to improve the measurement sensitivity, is also attempted in this study. The MWNT field emission ionization gauge is demonstrated in Fig. 3b, where the MWNT source is mounted 2 mm above the anode grid. The top source layout benefits the elongation of the electron trajectory to improve the gauge sensitivity [17, 26, 27]. The MWNT source exhibits excellent emission properties while integrating into the gauge. As shown in Fig. 4a, the emission current of 74 μA is reached under the gate potential of 350 V with the electron transmittance (anode current I_e_/cathode current I_c_) of about 60 %. Beyond the physical structure and the extraction field, the anode potential brings about significant impacts on the electron transmittance. The transmittance (*I*_e_/*I*_c_) increases from 38.2 to 74.4 % when the anode/gate potential ratio *V*_a_/*V*_g_ varies from 1:1 to 2:1, as shown in Fig. 4b. The anode potential could penetrate through the gate grid to enhance the extraction field and also assists the electron flying off the gate grid. The electron source exhibits good emission stability behaviors in wide pressure range. However, the current fluctuation increases to ±4.24 % in 2.59 × 10^−9^ Torr vacuum, comparing with ±2.72 % in 2.48 × 10^−10^ Torr, which is believed to be related to the gas adsorption, such as nitrogen and hydrogen [16, 28].

**Fig. 5:**
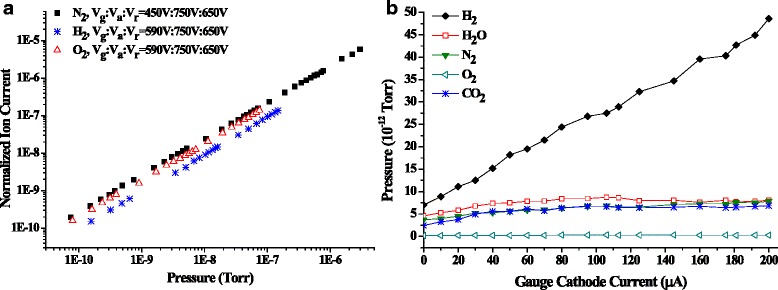
MWNT gauge performances. **a** Measurement linearities in nitrogen, hydrogen, and oxygen environments. **b** Partial pressure variations with the increase of emission current

The working principle of the ionization gauge is described by the relationship [29]: *I*_i_/*I*_e_ = K*P, where *I*_i_ is the ion current, *I*_e_ is the electron current (for the MWNT gauge, *I*_e_ is the anode current), *P* is the pressure, *K* is the gauge sensitivity factor, and the ratio *I*_i_/*I*_e_ is defined as the normalized ion current. *K* depends on the structural and the electric properties of the gauge. Figure 5a shows the calibration curves between the normalized ion current and pressure in different gas environments. The MWNT gauge presents excellent measurement linearity from 10^−11^ to 10^−6^ Torr with the sensitivity factor of about 2.22 Torr^−1^ for nitrogen. To our knowledge, this is the lowest lower pressure measurement limit reached in the reports for the CNT field emission ionization gauges. This excellent UHV measurement performance is benefited from the advantageous MWNT grown technique and the electron source design, which enable the low outgassings from the electron source. Further extension of the lower pressure measurement limit is subject to the accurate measurements of very weak ion current signals. The MWNT gauge also shows good measurement linearities for hydrogen and oxygen gases. Due to lower ionization efficiency, the sensitivity factor with hydrogen is about one third of the value with nitrogen. The oxygen calibration curve is slightly lower than nitrogen curve owing to the similarity of the ionization efficiencies between two gas species [30–32].

The gauge outgassing property, including the electron stimulated desorption (ESD) effect, plays a key role for the measurement accuracy and the XHV feasibility. This MWNT ionization gauge exhibits excellent low outgassing behaviors, leading to the superior measurement performance. Under the emission current of 100 μA, the system pressure may go up about 2 × 10^−10^ Torr in the 10^−11^ Torr vacuum. After the electrode cleaning from the higher emission flashing, i.e., 200 μA for a couple of minutes, the pressure rise could be smaller than 7 × 10^−11^ Torr. The electron bombardment cleans the surface adsorptions effectively. The outgassing properties for different gas species are further investigated. Figure 5b shows the partial pressure variations with increasing the emission current up to 200 μA. Hydrogen accounts for the major gas kind, which increases roughly linearly from 7.01 × 10^−12^ to 4.86 × 10^−11^ Torr. The steady rise of the hydrogen partial pressure is believed to be related to H_2_ desorptions from interiors of the metal electrodes due to the temperature rise by electron bombardments [33, 34]. With increasing the emission to 106 μA, partial pressures of H_2_O, N_2_, CO_2_, and O_2_ increase from 4.57 × 10^−12^ to 8.72 × 10^−12^ Torr (91 %), 3.69 × 10^−12^ to 6.78 × 10^−12^ Torr (84 %), 2.46 × 10^−12^ to 6.68 × 10^−12^ Torr, and 1.47 × 10^−13^ to 3.0 × 10^−13^ Torr, respectively, and reach stable states or drop afterwards. The gentle ascents for these four gases before 100 μA are ascribed to surface outgassings. The low outgassing performance, which enables the extension of the lower pressure limit to 10^−11^ Torr level, is attributed to several aspects, including the mechanical assembly of the cathode consisting of low outgassing materials, low extraction potentials, and the direct growth of MWNT emitters without extra gas adsorption materials.

**Fig. 6:**
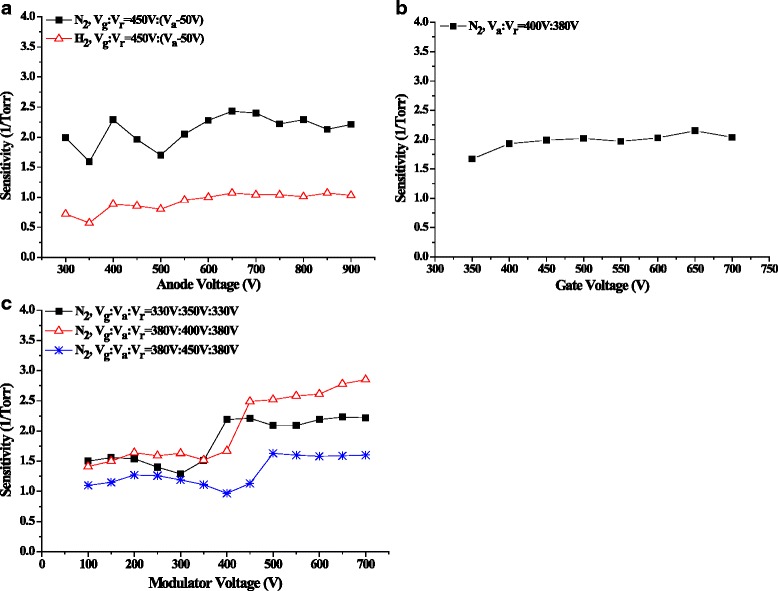
Sensitivity behaviors of the MWNT gauge. **a** Sensitivity and anode potential relations for nitrogen and hydrogen. **b** Sensitivity and gate potential relation for nitrogen. **c** Sensitivity and the modulator potential relations

The measurement sensitivity is determined by several factors, including the gas species, the gauge structure, and the electrode potentials. After balancing several key characteristic properties, including high ionization efficiency and low outgassing rates, the thermionic cathode ionization gauges are commonly designed to maintain the electron energies at around 150~200 V [3, 32, 35]. For the field emission based gauge, the electron energies are normally higher due to the existence of high electron extraction field [16, 18, 36]. The variations of the sensitivity factor *K* with the increases of the anode and the gate potentials are illustrated in Fig. 6. When varying the anode potential, the *K* for nitrogen bumps up and down and reaches the maximum value of 2.43 Torr^−1^ at 650 V, and *K* with hydrogen changes in the same rhythm. The fluctuations of the sensitivities could be related to the electron trajectories which are influenced by the electric field distribution [26]. While keeping the anode potential constant and raising the gate potential, *K* also rises and reaches 2.15 Torr^−1^ at 650 V. The increase of sensitivity factor with the potential is believed to be related to the elongations of the electron trajectories [20, 26, 35], even though the ionization efficiencies of major air components, i.e., N_2_, H_2_, and O_2_, drop beyond energies of 200 eV.

The improvement of the sensitivity factor is particularly important to extend the measurement down to UHV/XHV range where ion collection currents are normally extremely small. To evaluate the sensitivity performance by modulating the electron energy, the role of a mesh modulator with 92.2 % physical transparency is investigated. As shown in Fig. 6c, the relations between *K* and the modulator potential are tested for three anode potentials of 350, 400, and 450 V, respectively. With increasing the modulator potential from 100 to 700 V, the sensitivity factor varies roughly in two plateaus, and a transition leap occurs when the modulator potential is equal to and 50 V higher than the anode potential. Further increase of the modulator potential does not improve the *K* factor significantly. This leap is most probably related to the prolongations of the electron trajectories because a slightly higher modulator potential assists the electrons move back and forth around the top of the anode grid. Under the anode potential of 400 V, *K* reaches 2.85 Torr^−1^, 20 % higher than the value without the modulator. Liu et al. also demonstrated the sensitivity improvement by a shield electrode for the CNT BA gauge [37]. Therefore, the electron energy modulation enhances the gauge measurement sensitivity effectively.

## Conclusions

In summary, we have developed a carbon nanotube field emission ionization gauge after growing MWNTs directly on stainless steel substrates. The MWNT cathode presents excellent emission J-E and stability performances after the substrate anodization. The MWNT gauge demonstrates excellent measurement linearity in wide pressure range with the lower measurement limit down to 10^−11^ Torr, attributed largely to low outgassings due to the direct growth of MWNTs. The modulation of the electron energy benefits the improvement of the measurement performance. This gauge shows potential for UHV/XHV pressure measurements.

## Additional file

Additional file 1:
**Additional information.** Substrate surface morphology variations after anodization and the residual gas mass spectroscopy of the experimental system. (DOCX 322 kb)
