# Supplementary material for: Wide-range Vacuum Measurements from MWNT Field Emitters Grown Directly on Stainless Steel Substrates
Source: Nanoscale Res Lett. 2016 Jan 6;11:5. doi: 10.1186/s11671-015-1207-6 (PMC4703604; doi:10.1186/s11671-015-1207-6)
Supplement: Additional file 1: — Additional information. Substrate surface morphology variations after anodization and the residual gas mass spectroscopy of the experimental system. (DOCX 322 kb) [file 11671_2015_1207_MOESM1_ESM.docx]

**Additional Information**

Wide-range vacuum measurements from MWNT field emitters grown directly on stainless steel substrates

Jian Zhang^1^, Detian Li^1,2*^, Yangyang Zhao^1^, Yongjun Cheng^2^ , Changkun Dong^1,2+^

^1^Institute of Micro-nano Structures & Optoelectronics

Wenzhou University, Chashan University Town, Wenzhou, China

^2^Science and Technology on Vacuum & Cryogenics Technology and Physics Laboratory,

Lanzhou Institution of Physics, Lanzhou, China

*Email: lidetian@hotmail.com, ^+^Email: [dck@wzu.edu.cn](mailto:dck@wzu.edu.cn)

**Index**

**AI-1. Comparison of surface morphologies between regular and anodization substrates.**

**AI-2. Mass spectroscopy of the experimental system at 5.7×10^-8^ Pa.**

**AI-1. Comparison of surface morphologies between regular and anodization substrates.**

**
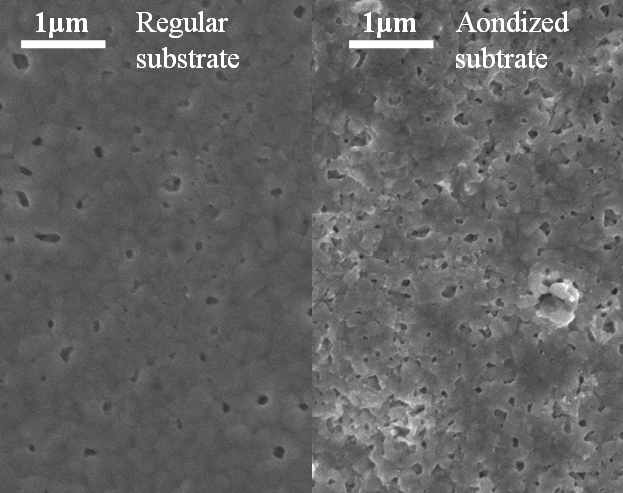
**

**AI-2. Mass spectroscopy of the experimental system at 5.7×10^-8^ Pa.**

**
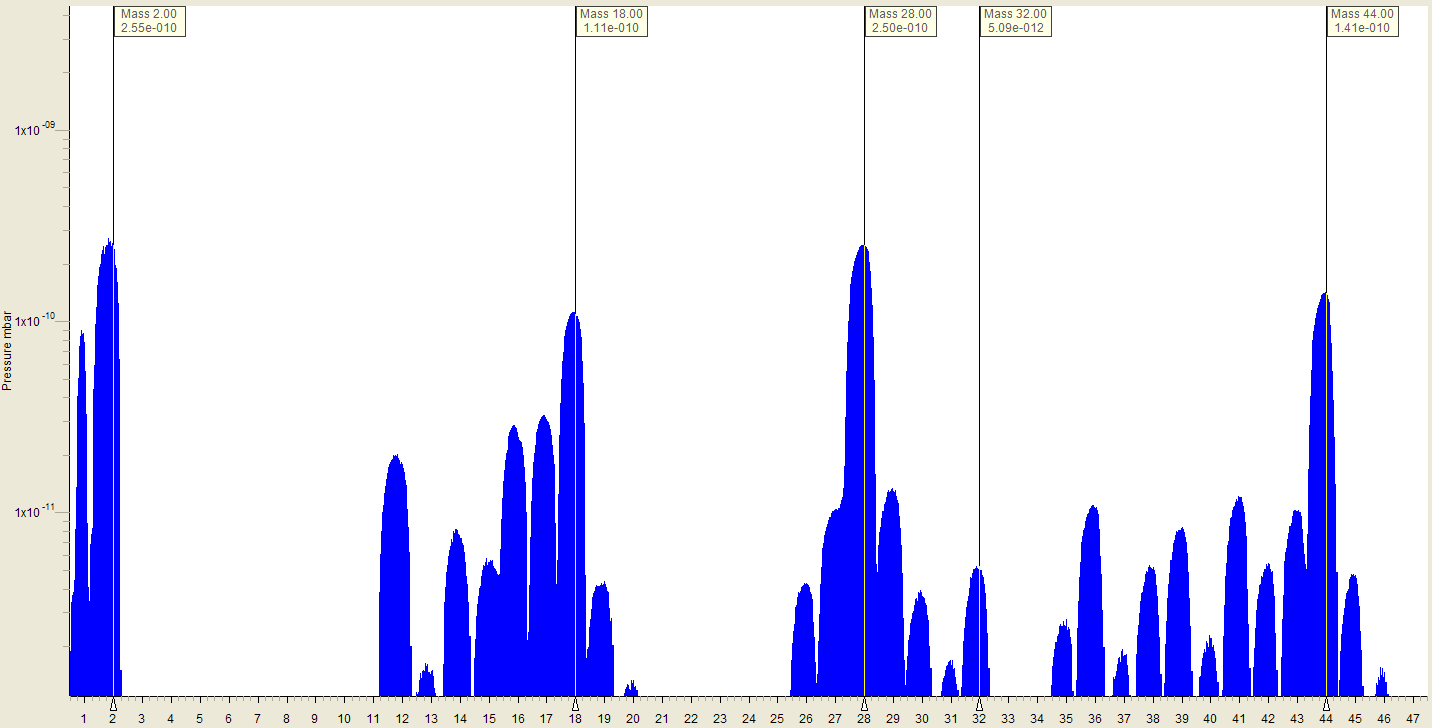
**
